# Large inferior vena cava thrombosis in a 25-year-old female patient: A case report

**DOI:** 10.1097/MD.0000000000040442

**Published:** 2024-11-08

**Authors:** Jiyoul Yang, Dae-Hwan Bae, Bumhee Yang, Minjung Bak, Yoon Mi Shin

**Affiliations:** a Division of Pulmonary and Critical Care Medicine, Department of Internal Medicine, Chungbuk National University Hospital, Chungbuk National University College of Medicine, Cheongju, Republic of Korea; b Division of Cardiology, Department of Internal Medicine, Bucheon Sejong Hospital, Bucheon, Republic of Korea; c Division of Cardiology, Department of Medicine, Heart Vascular Stroke Institute, Samsung Medical Center, Sungkyunkwan University School of Medicine, Seoul, Republic of Korea.

**Keywords:** inferior vena cava, intractable, systemic lupus erythematosus, thrombolysis, venous thromboembolism

## Abstract

**Rationale:**

The incidence of venous thromboembolism is increasing, and it is more common in older than in younger patients. Inferior vena cava (IVC) thrombosis is a rare subtype of deep vein thrombosis, and it is associated with a high incidence of arterial and venous thrombosis in patients with systemic lupus erythematosus (SLE). We present the case of a 25-year-old female patient with a large IVC thrombosis caused by SLE that was intractable to thrombolytic therapy.

**Patient concerns:**

A 25-year-old previously healthy female patient presented to the emergency department with a 5-day history of fever.

**Diagnoses:**

Her computed tomography revealed a large thrombus with an approximate length 25 cm extending from the suprarenal IVC to the right common iliac vein and the left external and internal iliac veins.

**Interventions:**

We decided to perform intravenous thrombolysis (alteplase 65 mg) because of the massive thrombus burden followed by an infusion of intravenous heparin. However, the size of the thrombus did not showed reduction 3 days after treatment. For the next step, the catheter-directed thrombolysis (CDT) was performed; however, no changes were observed.

**Outcomes:**

She was diagnosed with SLE, and the large IVC thrombus gradually improved after primary treatment for SLE, including immunosuppressive drugs combined with anticoagulant.

**Lessons:**

We found a large IVC thrombus secondary to SLE in a young female patient. In this case, systemic thrombolysis or CDT did not improve the thrombus. The thrombus only improved after immunosuppressive treatment for SLE. Our case highlights the importance of treatment for etiologic disease in an intractable thrombosis developed by provoked cause.

## 1. Introduction

The inferior vena cava (IVC) is a largest retroperitoneal vein formed by the confluence of the right and left common iliac veins. The IVC is responsible for transporting deoxygenated blood for the lower extremities, abdomen, pelvis and back to the right atrium.^[1]^ Deep vein thrombosis (DVT) is an obstructive disease with interfering venous reflux mechanism.^[2,3]^ The prevalence of involvement at specific anatomical sites in venous thrombosis is as follows: distal veins 40%, popliteal veins 16%, femoral veins 20%, common femoral veins 20%, and iliac veins 4%.^[4]^ Thrombosis in the IVC is relatively rare, accounting for approximately 1.3% of DVT cases identified in the United States between 1979 and 2005.^[5,6]^ Symptoms of DVT range from asymptomatic to massive edema and cyanosis with impending venous gangrene.^[4]^ The etiologies of IVC thrombosis have been classified into 3 categories: association between IVC thrombosis and venous thromboembolism; idiopathic or primary IVC thrombosis; provoked/secondary IVC thrombosis. The provoked or secondary IVC thrombosis category was subdivided into 2 according to outflow obstruction: secondary or provoked IVC thrombosis without outflow obstruction and IVC thrombosis with outflow obstruction.^[7]^

Systemic lupus erythematosus (SLE) is a chronic autoimmune disease with diverse clinical manifestations that predominantly develops in young women.^[8]^ The incidence of arterial and venous thrombosis in patients with SLE is high, at 36.3 per 1000 patient-years.^[9,10]^ The mechanism of thrombosis in SLE is predominantly explained by its association with antiphospholipid antibodies.^[11–13]^ Several cases of IVC thrombosis accompanied by renal vein thrombosis have been reported in patients with lupus nephritis,^[14–16]^ but there have been no case reports of a large IVC thrombosis in lupus nephritis which we experienced. We present a case of a 25-year-old female patient with a large IVC thrombosis caused by SLE.

## 2. Case presentation

A previously healthy 25-year-old female patient presented to the emergency department with a 5-day history of fever. Petechia-like hemorrhagic spots were observed on both hands, and foamy urine was present for 1 week. The fever was predominantly aggravated at night, and there was bilateral edema of the lower extremities, which was more pronounced in the left leg for 5 days. She had no usual medications, history of travel or vaccination within the past month. None of her family members had hematological disorders. She was a nonsmoker and rarely consumed alcohol.

**Figure 1.**
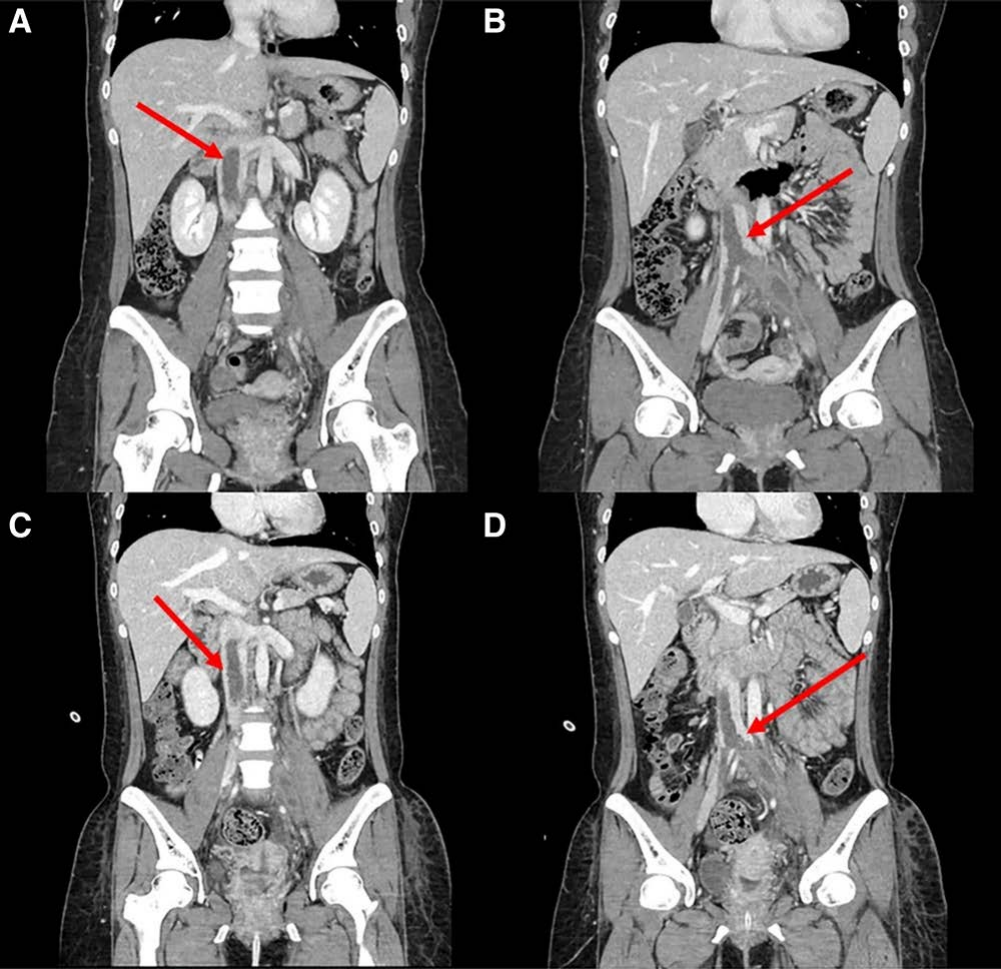
The initial abdominal CT shows a large IVC thrombus with a long segment. Red arrow lines show thrombus in the IVC. (A, B) CT scan was obtained before systemic thrombolysis. (C, D) CT scan was obtained 3 days after systemic thrombolysis. There was no significant change in the size of the thrombus before and after systemic thrombolysis. CT = computed tomography, IVC = inferior vena cava.

Physical examination showed an initial blood pressure of 120/80 mm Hg, heart rate of 88 beats per minute, respiratory rate of 20 breaths per minute, and body temperature of 36.5°C. After 10 hours in the emergency department, she developed a fever of 38.0°C and tachycardia. Her heart and breathing sounds were normal. Tenderness was observed on palpation of both lower extremities. The chest and abdominal computed tomography (CT) were performed. Her chest CT revealed a mild bilateral pleural effusion but there was no pulmonary embolism. Abdominal CT showed a large thrombus with an approximate length of 25 cm extending from the suprarenal IVC to the right common iliac vein and the left external and internal iliac veins (Fig. 1). The left common iliac vein showed focal stenosis with perivenous fat infiltration. Initial laboratory tests revealed mild leukocytosis (10.3 × 10^3^/µL, normal 4.0–10.0 × 10^3^/µL), normal platelet count, and normal hemoglobin concentration. The high-sensitivity C-reactive protein concentration was 4.88 mg/dL (normal 0–0.3 mg/dL), but the procalcitonin concentration was within the normal range at <0.05 ng/mL. The fibrinogen degradation product and D-dimer concentration were 232.5 µg/mL (normal 0–5 µg/mL) and 80.0 µg/mL (normal 0–0.5 µg/mL), respectively. The fibrinogen concentration was 812.4 mg/dL (normal, 170–410 mg/dL), and the anti-thrombin III level was 54.2% (normal, 75–125%). Urinalysis revealed proteinuria and hematuria.

The patient was admitted to the intensive care unit for continuous monitoring. She was hemodynamically stable, but we decided to perform intravenous thrombolysis (alteplase 65 mg) because of the high thrombus burden, followed by the infusion of intravenous heparin. Antibiotics were administered because an infection could not be completely ruled out. Laboratory tests were initially performed to determine the etiology of DVT, but we could not obtain the results during her admission. The follow up CT was performed 3 days after admission, but there was no significant change in the extent of thrombosis. Therefore, we planned catheter-based thrombectomy or catheter-directed thrombolysis (CDT), but the patient’s family wanted to be transferred to the Samsung Medical Center 4 days after admission.

**Figure 2.**
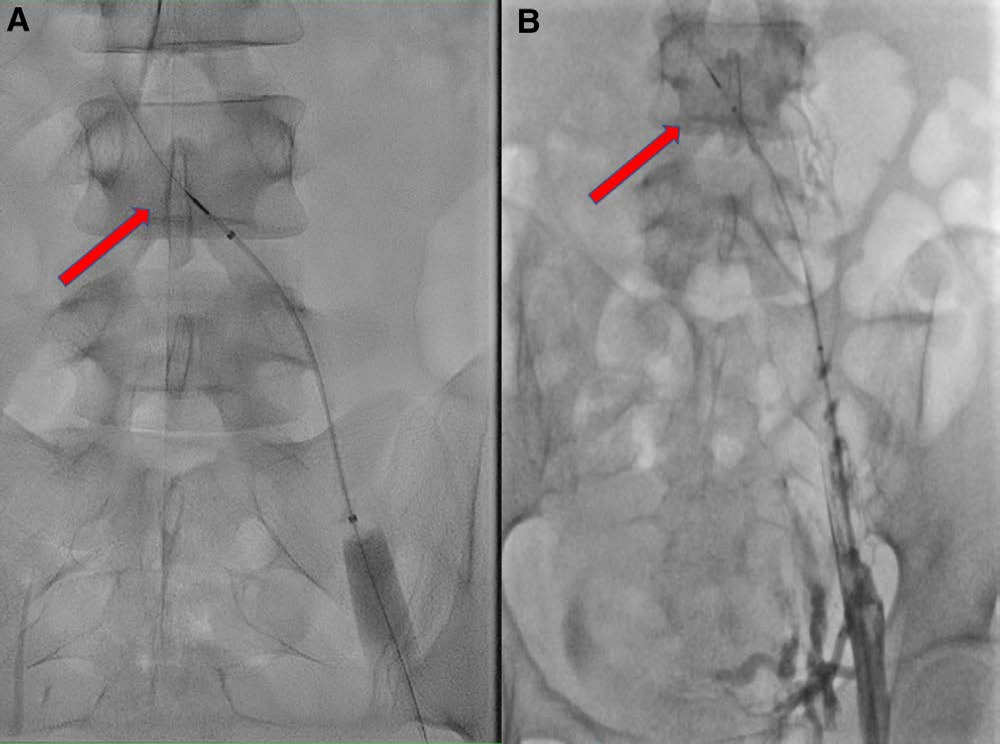
Venogram before and 24 hours after CDT. The red arrow lines indicate a thrombus in the IVC. When comparing the venogram before CDT (A) and the venogram 24 hours after CDT (B), it can be seen that there is little difference in the burden of IVC thrombosis. CDT = catheter-directed thrombolysis, IVC = inferior vena cava.

After arriving at Samsung Medical Center, the patient was managed with IVC filter insertion at the suprarenal IVC (above the IVC thrombus) and CDT. CDT was performed twice, every 12 hours. Alteplase was used for thrombolytic agent during CDT. There was no difference in filling defects of the venogram 24 hours after CDT compared to before CDT (Fig. 2). The patient was performed duplex ultrasound 1 week after CDT. The duplex ultrasound showed total thrombotic occlusion from Lt. common iliac vein to the posterior tibial vein.

The initial results of laboratory findings for autoimmune diseases were revealed after her discharge. The results were positive for the anti-nuclear antibody at a 1:80 dilution, anti-double stranded deoxyribonucleic acid antibody (9.37 IU/mL), and lupus anticoagulant (1.91). Complements 3, 4 and antiphospholipid antibodies (APL) were within the normal range. Her results were negative for the myeloperoxidase and proteinase 3. The concentrations of proteins C, S and factor V were within normal limits.

Three days after admission to another hospital, methylprednisolone 1 mg/kg was administered under the suspicion of an autoimmune disorder, and the patient underwent a kidney biopsy to diagnose lupus nephritis 7 days after being transfer. Two days after the kidney biopsy, the pathological report suggested lupus nephritis type V, and the patient was prescribed prednisolone 60 mg once daily, tacrolimus 3 mg once daily, hydroxychloroquine 300 mg once daily, and apixaban 5 mg twice daily on discharge from the hospital. The patient was treated with oral anticoagulation (apixaban) for 1 year after discharge.

**Figure 3.**
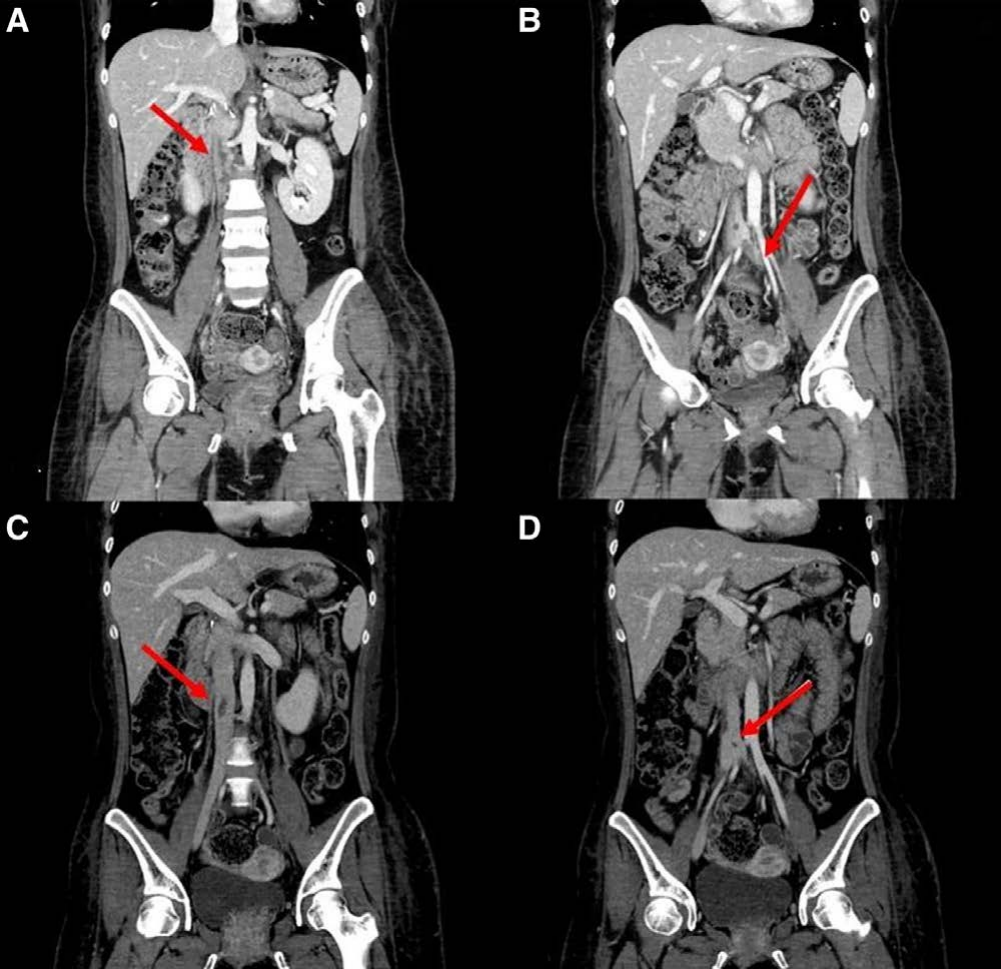
(A, B, C, D) The follow up abdominal CT scans. The red arrow lines indicate a thrombus in the IVC. CT scans were obtained 2 months after admission and show a reduced burden of the IVC thrombus. CT = computed tomography, IVC = inferior vena cava.

Her follow up abdominal CT at 1 month showed a minimal decrease in the size of the IVC thrombus. The last abdominal CT performed 2 months later revealed a significant decrease of the IVC thrombus (Fig. 3).

## 3. Discussion

Thrombosis of the IVC is rare, especially in young patients^[17,18]^ This patient developed thrombosis secondary to SLE and the length of the thrombus was 25 cm, which was the largest IVC thrombus that we have experienced until now.

In SLE, there are several mechanisms by which thrombus formation can occur. APL can activate platelets and interact with endothelial cells, contributing to thrombogenesis. Chronic inflammation leads to the release of cytokines and inflammatory mediators, which can damage endothelial cells, promoting platelet adhesion to the damaged endothelium and subsequent thrombus formation. Additionally, antigen-antibody complexes can form, leading to vascular damage and thrombogenesis. Impairment of anticoagulant mechanisms, such as reductions in proteins C and S, can also contribute to thrombosis.^[11]^ In this patient, APL were negative, and the levels of anticoagulant proteins were within normal ranges. Therefore, it is likely that thrombus formation was primarily due to vascular damage caused by antigen-antibody complexes and chronic inflammation associated with SLE.

The clinical manifestations of IVC thrombosis vary with the anatomical level of the thrombosis and the degree of occlusion of the IVC. If the thrombus is confined to the IVC, its clinical presentation may be vague because collateral pathways form along the posterior abdominal wall.^[5]^ For this reason, isolated IVC thrombosis may be difficult to detect. The clinical presentation of the patient, including fever, petechiae, foamy urine, and bilateral lower-extremity edema was consistent with the manifestations of renal involvement in SLE and it might also be related to this thrombosis.

In this case, the large IVC thrombus was intractable to systemic thrombolysis and CDT. The treatment of choice for IVC thrombosis is anticoagulation with thrombolysis,^[7,19,20]^ but it yielded no reduction of the thrombus in this patient. The large IVC thrombus located across this long segment gradually improved after primary treatment for SLE, such as immunosuppressive drugs combined with anticoagulants.

## 4. Conclusion

We found a large IVC thrombus secondary to SLE in a young female patient. The thrombus did not improve with systemic or CDT. The thrombus only improved after immunosuppressive treatment for SLE. Our case highlights the importance of treatment for etiologic disease in an intractable thrombosis developed by provoked cause.

## Author contributions

**Conceptualization:** Bumhee Yang, Yoon Mi Shin.

**Data curation:** Dae-Hwan Bae, Minjung Bak, Yoon Mi Shin.

**Formal analysis:** Jiyoul Yang, Bumhee Yang.

**Investigation:** Jiyoul Yang, Dae-Hwan Bae, Bumhee Yang.

**Methodology:** Yoon Mi Shin.

**Project administration:** Yoon Mi Shin.

**Resources:** Bumhee Yang, Minjung Bak, Yoon Mi Shin.

**Supervision:** Yoon Mi Shin.

**Validation:** Dae-Hwan Bae.

**Writing – original draft:** Jiyoul Yang.

**Writing – review & editing:** Dae-Hwan Bae, Yoon Mi Shin.

## References

[1] Li SJ Cai H Cai J. The inferior vena cava: anatomical variants and acquired pathologies. Insights Imaging. 2021;12:123. 34460015 10.1186/s13244-021-01066-7 PMC8405820

[2] Huang Y Lyu J Jin R Liu D Zhang Y Xie H. Association between blood lipid levels and lower extremity deep venous thrombosis: a population-based cohort study. Clin Appl Thromb Hemost. 2022;28:10760296221121282. 36189865 10.1177/10760296221121282 PMC9530559

[3] Waheed SM Kudaravalli P Hotwagner DT. Deep Vein Thrombosis. In: StatPearls. Treasure Island (FL): StatPearls Publishing; 2024. 29939530

[4] Stubbs MJ Mouyis M Thomas M. Deep vein thrombosis. BMJ. 2018;360:k351. 29472180 10.1136/bmj.k351

[5] McAree BJ O’Donnell ME Fitzmaurice GJ Reid JA Spence RA Lee B. Inferior vena cava thrombosis: a review of current practice. Vasc Med. 2013;18:32–43. 23439778 10.1177/1358863X12471967

[6] Stein PD Matta F Yaekoub AY. Incidence of vena cava thrombosis in the United States. Am J Cardiol. 2008;102:927–9. 18805124 10.1016/j.amjcard.2008.05.046

[7] Shi W Dowell JD. Etiology and treatment of acute inferior vena cava thrombosis. Thromb Res. 2017;149:9–16. 27865097 10.1016/j.thromres.2016.07.010

[8] Fanouriakis A Kostopoulou M Alunno A. Update οn the diagnosis and management of systemic lupus erythematosus. Ann Rheum Dis. 2021;80:14–25. 33051219 10.1136/annrheumdis-2020-218272

[9] Petri M. Thrombosis and systemic lupus erythematosus: the Hopkins Lupus Cohort perspective. Scand J Rheumatol. 1996;25:191–3. 8792794 10.3109/03009749609069986

[10] Romero-Díaz J García-Sosa I Sánchez-Guerrero J. Thrombosis in systemic lupus erythematosus and other autoimmune diseases of recent onset. J Rheumatol. 2009;36:68–75. 19012362 10.3899/jrheum.071244

[11] Al-Homood IA. Thrombosis in systemic lupus erythematosus: a review article. ISRN Rheumatol. 2012;2012:428269. 22900201 10.5402/2012/428269 PMC3413961

[12] de Groot PG de Laat B. Mechanisms of thrombosis in systemic lupus erythematosus and antiphospholipid syndrome. Best Pract Res Clin Rheumatol. 2017;31:334–41. 29224675 10.1016/j.berh.2017.09.008

[13] Bazzan M Vaccarino A Marletto F. Systemic lupus erythematosus and thrombosis. Thromb J. 2015;13:16. 25908929 10.1186/s12959-015-0043-3 PMC4407320

[14] Appel GB Williams GS Meltzer JI Pirani CL. Renal vein thrombosis, nephrotic syndrome, and systemic lupus erythematosus: an association in four cases. Ann Intern Med. 1976;85:310–7. 962221 10.7326/0003-4819-85-3-310

[15] Hamilton CR Jr Tumulty PA. Thrombosis of renal veins and inferior vena cava complicating lupus nephritis. JAMA. 1968;206:2315–7. 5696091

[16] Mintz G Acevedo-Vázquez E Gutiérrez-Espinosa G Avelar-Garnica F. Renal vein thrombosis and inferior vena cava thrombosis in systemic lupus erythematosus. Frequency and risk factors. Arthritis Rheum. 1984;27:539–44. 6721886 10.1002/art.1780270509

[17] White RH. The epidemiology of venous thromboembolism. Circulation. 2003;107(23 Suppl 1):I4–8. 12814979 10.1161/01.CIR.0000078468.11849.66

[18] Alkhouli M Morad M Narins CR Raza F Bashir R. Comparative outcomes of catheter-directed thrombolysis plus anticoagulation versus anticoagulation alone in the treatment of inferior vena caval thrombosis. Circ Cardiovasc Interv. 2015;8:e001882. 25663321 10.1161/CIRCINTERVENTIONS.114.001882

[19] Alkhouli M Bashir R Raza F Morad M Lewandowski R Moukarbel G. Inferior vena cava thrombosis. JACC Cardiovasc Interv. 2016;9:629–43. 26952909 10.1016/j.jcin.2015.12.268

[20] Golowa Y Warhit M Matsunaga F Cynamon J. Catheter directed interventions for inferior vena cava thrombosis. Cardiovasc Diagn Ther. 2016;6:612–22. 28123981 10.21037/cdt.2016.11.09 PMC5220197

